# The current status and improvement directions of legal rules regarding Chinese national gene banks for farm animal genetic resources

**DOI:** 10.3389/fgene.2024.1413625

**Published:** 2024-10-09

**Authors:** Qi Chen, Chien Te Fan, Hongtao Jiao

**Affiliations:** ^1^ International Intercollegiate Ph.D. Program, Institute of Law for Science and Technology, National Tsing Hua University, Hsinchu, Taiwan; ^2^ Institute of Law for Science and Technology, National Tsing Hua University, Hsinchu, Taiwan; ^3^ Institute of Law for Science and Technology, Huazhong University of Science and Technology, Wuhan, China

**Keywords:** biodiversity, gene banks, farm animal genetic resources, utilization, benefit-sharing, Chinese law

## Abstract

A gene bank for farm animal genetic resources (FAGR) is an important facility for the diversity conservation of FAGR. The primary purpose of gene banks for FAGR is the reconstruction of those breeds. With the advent of the post-genomic era, gene banks for FAGR have increasingly become an infrastructure for the support of livestock research and animal breeding, and a platform for international research collaboration. China is one of the richest countries in the world in terms of FAGR. Chinese National Gene Banks for FAGR (CNGBFs) have an important legal status and play an important function in the Chinese FAGR protection system. In this paper, we reviewed the current situation of CNGBFs construction, and systematically collected and analyzed legal rules related to CNGBFs. As results, those legal rules were categorized into two types: (a) organization and management, (b) activities. We summarized problems existing in the current legal rules for CNGBFs from three levels: institution, practical operation, and digital development. Improvement directions of legal rules regarding CNGBFs are proposed. They include clarifying the utilization function of CNGBFs and the ownership of FAGR in CNGBFs. Moreover, improving the mechanism of administrative management, rules on domestic and international access and benefit-sharing, and the system of digital sequence information management are also suggested. The improvements in those legal rules will contribute to the appropriate utilization of Chinese FAGR and international collaborations.

## 1 Introduction

The diversity conservation of farm animal genetic resources (FAGR) is a global concern as these resources are of significant importance to the environment, genetics, society, economy, medicine, science, education, and cultural spirituality (FAO, 2007b). In 1992, the *Convention on Biological Diversity* (*CBD*) (Secretariat of the Convention on Biological Diversity, 1992) explicitly recognized “domesticated species” as an important component of global biodiversity and stipulated the obligation of contracting parties to conserve biodiversity (FAO, 1998; Articles 2 and 6 of the CBD). In 2007, the international community specifically reached the *Interlaken Declaration on Animal Genetic Resources* (the *Interlaken Declaration*) (FAO, 2007b) and the *Global Plan of Action for Animal Genetic Resources* (the *Global Plan*) (FAO, 2007a) to conserve FAGR. Among them, the *Global Plan* explicitly requires countries to adopt a combination of *in situ* conservation and *ex situ* conservation (Strategic Priority Area 3). *In situ* conservation refers to “the maintenance of live populations of animals in their adaptive environment” (Henson, 1992). *Ex situ* conservation means “conservation away from the habitat and production systems where the resource developed” (FAO, 2012), including two types: maintenance of live animal populations and cryoconservation (FAO, 2012; FAO, 2007a). Cryoconservation involves the preservation of cells or tissues (such as embryos, semen, oocytes, somatic cells) or other biological samples (such as blood, DNA) that can be used to reconstruct live animals under cryogenic conditions (FAO, 2015; FAO, 2012) in a facility known as a “gene bank” (FAO, 2023).

The conservation of FAGR through gene banks has received increasing attention in the international community in recent years. According to the FAO *Second Report on the State of the World’s Animal Genetic Resources for Food and Agriculture*, as of 2015, gene banks for FAGR have been established and are operational in 58 countries around the world, and are scheduled in 46 countries (FAO, 2015). Gene banks for FAGR are highly valued internationally not only because of their ability to provide long-term storage of biological material from a wide range of species (FAO, 2012; Blackburn, 2009) in a cost-effective manner (Paiva et al., 2016), but also because of their capability of multiple uses. The primary use of a gene bank is to reconstruct extinct species of livestock and poultry and to back up genetic diversity. Besides, a gene bank can provide biological materials for breeding and genetic research (FAO, 2012). For example, the owners of rare cattle breeds used the genetic resources from gene banks in the United States to restore genetic variations for the two rare cattle breeds, Dexter and Milking Shorthorn (Blackburn et al., 2019). Also, the Dutch gene bank provided genetic information of rare local breeds, facilitating the study for the genetic background of milk fatty acid composition (Groeneveld et al., 2016). With the establishment of gene banks for FAGR, it becomes crucial to develop laws and policies for the acquisition, release and exchange of genetic resources. Only in this way can the value of the genetic resources collected in gene banks be fully unleashed, thereby promoting the development of the livestock sector (Blackburn and Boettcher, 2010).

China is one of the countries with the richest FAGR in the world. According to the *National Breed List of FAGR (2021 Edition)*, China has 948 breeds, of which 546 are indigenous (Office of the Chinese National Commission for Farm Animal Genetic Resources, 2021). China attaches great importance to the conservation of FAGR (see Editorial Committee of Status of Chinese Farm Animal Genetic Resources, 2004). As a contracting party to the *CBD* and a participating country in the formulation of the *Global Plan* and the *Interlaken Declaration*, China actively fulfills its tasks and responsibilities in the conservation of FAGR. As early as 1994, the State Council of China promulgated the *Stock Animal Administration Regulations* (The State Council of China, 1994)^1^, which required competent administrative departments of the State Council and the people’s governments at the provincial level to set up conservation farms, protected areas, and gene banks for FAGR (Article 7). In 2005, China formally enacted *the Animal Husbandry Law of the People’s Republic of China* (the *Animal Husbandry Law*) (the Standing Committee of the National People’s Congress, 2005)^2^, which clearly stipulated three ways to conserve FAGR, namely, the establishment of conservation farms, protected areas, and gene banks [see Article 13 (1)]. These correspond to the internationally recognized methods of *ex situ in vivo* conservation, *in situ* conservation, and *ex situ* cryoconservation, respectively.

China also pays great attention to the construction of gene banks for FAGR. China has established gene banks for FAGR at the national and provincial levels. Chinese National Gene Banks for FAGR (CNGBFs) are responsible for the conservation of national conserved breeds^3^. Meanwhile, the conservation of provincial conserved breeds^4^ is undertaken by the respective provincial gene banks for FAGR. The conservation and appropriate utilization of FAGR is one of objectives of the *Animal Husbandry Law* (the Standing Committee of the National People’s Congress, 2022). However, from the perspective of legislative content, the *Animal Husbandry Law* and other legal norms have not provided sufficient support to realize the conservation and utilization functions of CNGBFs. Existing research lacks attention to this issue, and mainly focuses on legal rules related to the protection and utilization of plant genetic resources (see Zhang, 2012). There are some studies on the legal rules concerning access to and utilization of Chinese genetic resources (see Deng, 2018; Xue and Cai, 2009). Yet these studies do not focus on FAGR, nor do they pay attention to the legal issues specific to the construction of gene banks for FAGR.

The purpose of this paper is to examine the existing legal rules regarding CNGBFs and to make strategic thoughts on the improvement of these legal rules. This paper first presents the current situation of CNGBFs construction, and then reviews the sources of law regarding CNGBFs. Furthermore, this paper presents the legal rules regarding CNGBFs from the perspectives of (a) organization and management, and (b) activities. Finally, built upon the current status of the legal rules related to CNGBFs, this paper proposes their future direction for improvement.

## 2 The current situation of CNGBFs construction

**FIGURE 1:**
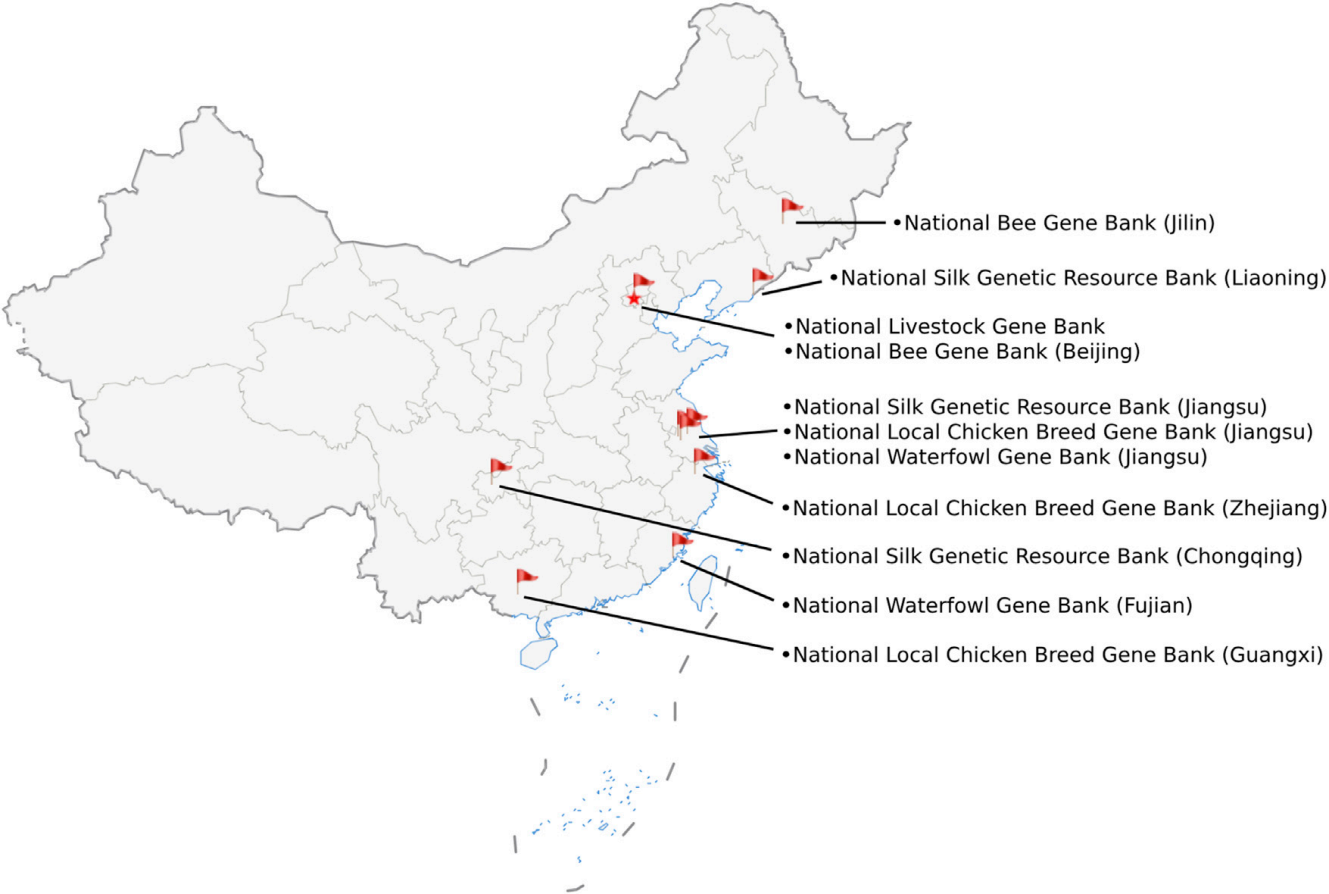
The locations of 11 supporting national gene banks in China.

In terms of the construction layout of CNGBFs, China aims to establish a core national farm animal germplasm resource bank, multiple supporting national gene banks (Chinese Ministry of Agriculture and Rural Affairs, 2021b), and their backups (Chinese Ministry of Agriculture and Rural Affairs, 2019c). The Institute of Animal Science of the Chinese Academy of Agricultural Sciences is currently constructing the core bank (see Table 1), aiming at the long-term strategic preservation of *in vitro* genetic materials (Chinese Ministry of Agriculture and Rural Affairs, 2021a). As of October 2023, Chinese Ministry of Agriculture and Rural Affairs of the State Council have approved a total of 11 supporting national gene banks (see Table 1). These gene banks have completed their construction and are distributed across eight regions, namely, Liaoning Province, Jilin Province, Zhejiang Province, Jiangsu Province, Fujian Province, Guangxi Zhuang Autonomous Region, as well as the two municipalities of Beijing and Chongqing (see Figure 1). Among these 11 supporting national gene banks, the National Livestock Gene Bank is in charge of the preservation of *in vitro* genetic materials. Other 10 supporting national gene banks are responsible for the preservation of live animals.

**TABLE 1:** Chinese national gene banks (As of October 2023).

Number	Confirmation time	Name	Construction institutions
1	Under construction	National farm animal germplasm resource bank	The Institute of Animal Science of the Chinese Academy of Agricultural Sciences
2	2023	National Silk Genetic Resource Bank (Liaoning)	Liaoning Institute of Seri cultural Science
3	2022	National Silk Genetic Resource Bank Jiangsu)	Seri cultural Research Institute of the Chinese Academy of Agricultural Sciences
4	2022	National Silk Genetic Resource Bank (Chongqing)	Southwest University
5	2021	National Livestock Gene Bank	National Animal Husbandry Station
6	2021	National Bee Gene Bank (Beijing)	Institute Apicultural Research of the Chinese Academy of Agricultural Sciences
7	2021	National Bee Gene Bank (Jilin)	Jilin Province Institute of Apicultural Science
8	2021	National Local Chicken Breed Gene Bank (Jiangsu)	Jiangsu Institute of Poultry Science
9	2021	National Local Chicken Breed Gene Bank (Zhejiang)	Zhejiang Guangda Agricultural Science and Technology Development Co., Ltd
10	2021	National Local Chicken Breed Gene Bank (Guangxi)	Guangxi Jinling Poultry Breeding Co., Ltd
11	2021	National Waterfowl Gene Bank (Jiangsu)	Jiangsu Vocational College of Agricultural Science and Technology
12	2021	National Waterfowl Gene Bank (Fujian)	Shishi Seed Industry Development Center

## 3 Legal rules for CNGBFs: the sources of law

At the international level, China has acceded to the following conventions and documents: the *CBD*, the *Global Plan*, the *Interlaken Declaration*, the *Nagoya Protocol on Access to Genetic Resources and the Fair and Equitable Sharing of Benefits Arising from their Utilization to the CBD* (the *Nagoya Protocol*) (Secretariat of the Convention on Biological Diversity, 2011), the *Agreement on Trade-Related Aspects of Intellectual Property Rights* (*TRIPS Agreement*) (WTO, 1994), the *Cartagena Protocol on Biosafety to the CBD* (Secretariat of the Convention on Biological Diversity, 2000), the *Convention for the Safeguarding of the Intangible Cultural Heritage* (UNESCO, 2003), and so forth.

At the domestic legislative level, the *Constitution* of China (Chinese National People’s Congress, 2018) does not contain direct provisions on the conservation, utilization, and benefit-sharing of FAGR. However, it does contain provisions on the conservation and utilization of natural resources (Article 9 of Chinese Constitution). In addition to the Constitution, provisions related to the conservation, utilization, and benefit-sharing of FAGR are dispersed among four levels of legal norms, which are formulated by the National People’s Congress and its Standing Committee, the State Council of China, departments under the State Council of China, and local legislatures with legislative power^5^.

## 4 Legal rules for CNGBFs: organization and management

**FIGURE 2:**
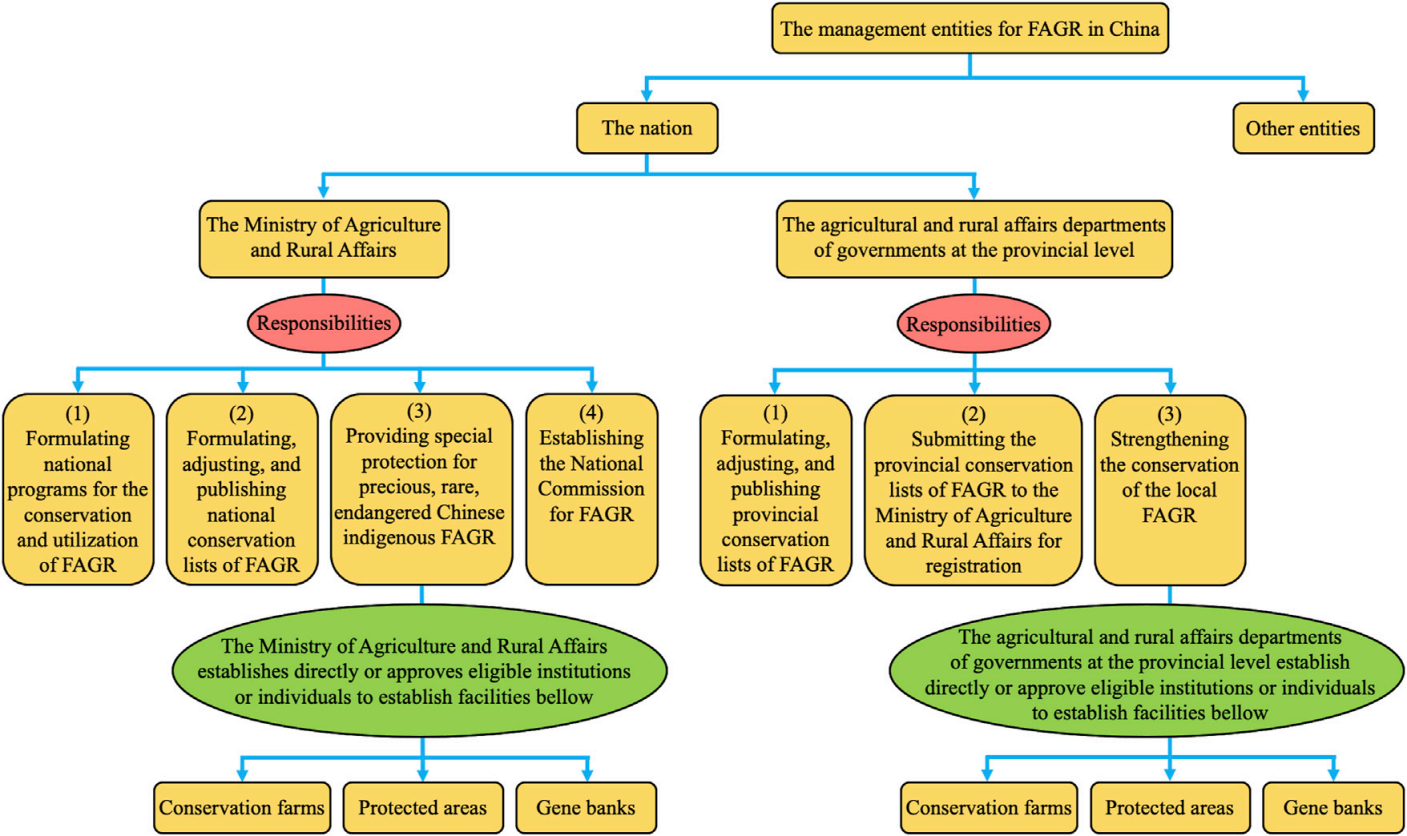
The management bodies and their responsibilities for FAGR in China. FAGR: farm animal genetic resources.

The *Animal Husbandry Law* is the basic law for the conservation of FAGR in China, and constructs a conservation system for FAGR (Figure 2). In terms of protection entities, they include the nation and multiple entities. However, they each assume different roles in the conservation system according to the provisions of the *Animal Husbandry Law*. Among them, the nation serves as the primary responsible entity for conserving FAGR [Article 10 (2) of the Animal Husbandry Law]. The *Animal Husbandry Law* establishes the responsibilities of the central and local governments in the protection of FAGR [see Articles 13, 14 (1), 11], and mandates the budget allocation for conserving these resources [Article 10 (1)]. Multiple entities are institutions and individuals. The *Animal Husbandry Law* does not impose mandatory obligations for conservation, but encourages and supports their participation in the conservation of FAGR [Article 10 (3)]. In terms of conservation methods, it encompasses conservation farms, protected areas, and gene banks. These facilities can be either directly established by the governments or eligible institutions or individuals approved by the governments [see Article 14 (1) of the Animal Husbandry Law; Article 9 of the Administration Measures for the Conservation Farms, Protected Areas, and Gene Banks for FAGR (Chinese Ministry of Agriculture, 2006 ^6^)].

**FIGURE 3:**
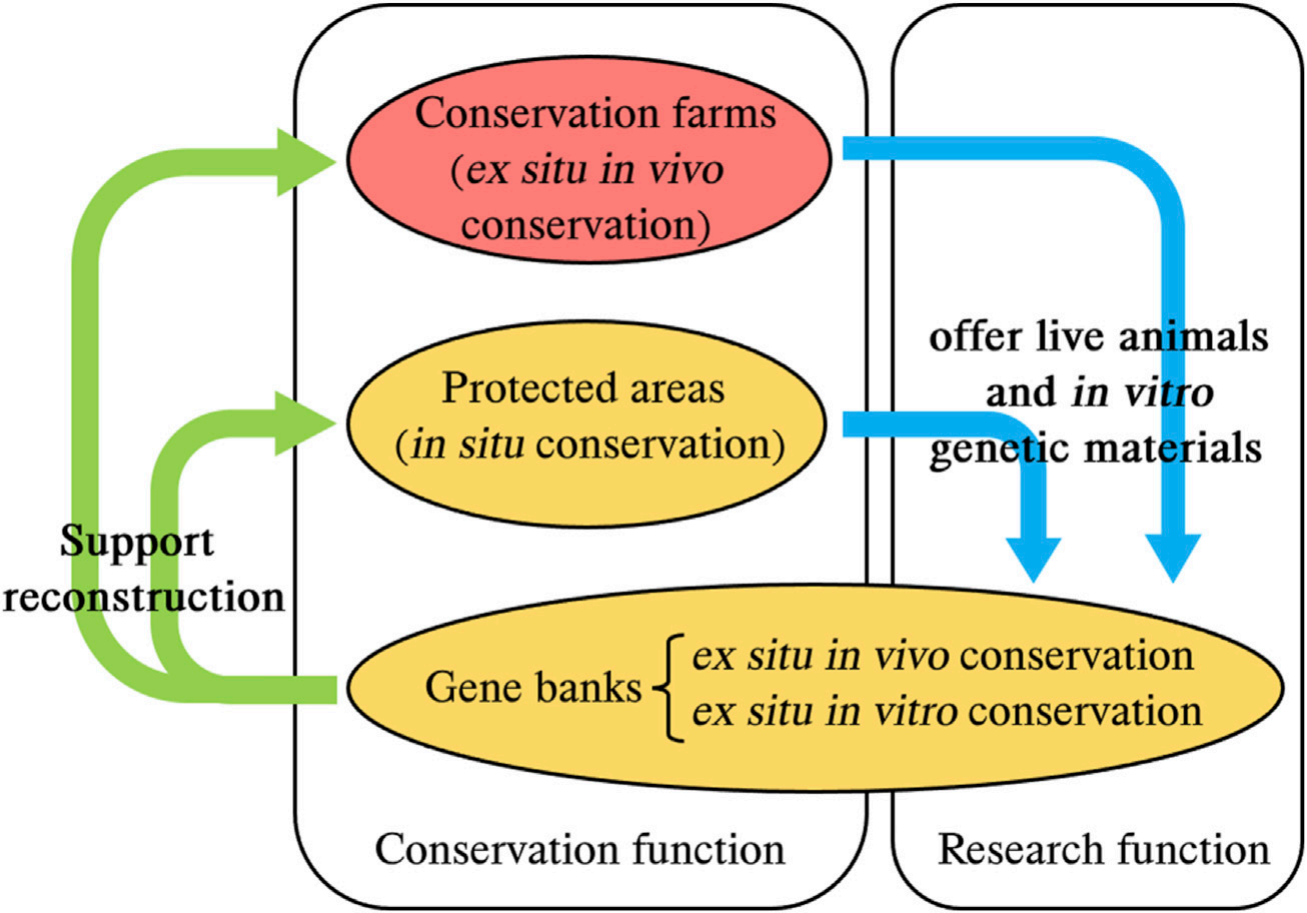
The roles and functions of gene banks for FAGR in China. The red represents the main method of conserving FAGR. The yellow represents the complementary method of conserving FAGR. This figure is drawn by authors after summarizing these materials (Chinese Ministry of Agriculture, 2011; Chinese Ministry of Agriculture and Rural Affairs, 2019a; Articles 5, 6, 21 of the Administration Measures for the Conservation Farms, Protected Areas, and Gene Banks for FAGR; Articles 10 (3) and 14 (1) of the Animal Husbandry Law). FAGR, farm animal genetic resources.

In the conservation system for FAGR, the gene bank holds a unique position and serves distinct functions (see Figure 3). It is particularly noteworthy that there are differences between Chinese legal provisions and the FAO *Guidelines on Cryoconservation of Animal Genetic Resources* (FAO, 2012) in terms of the scope of conservation in gene banks. The FAO Guidelines limits the scope of conservation in gene banks to *in vitro* genetic materials, excluding live animals; while Chinese laws include both *in vitro* genetic materials and live animals within the scope of conservation in gene banks (Article 21 of the Administration Measures for the Conservation Farms, Protected Areas, and Gene Banks for FAGR).

With regard to the management of CNGBFs, the *Administration Measures for the Conservation Farms, Protected Areas, and Gene Banks for FAGR* stipulates that CNGBFs shall be managed by the Ministry of Agriculture and Rural Affairs (Article 3), while the National Animal Husbandry Station is responsible for the specific administrative work (Article 4). However, in practice, CNGBFs are directly managed and operated by construction institutions, and the work of those institutions is subject to the supervision of the National Animal Husbandry Station (Articles 18, 19).

## 5 Legal rules for CNGBFs: activities

**FIGURE 4:**
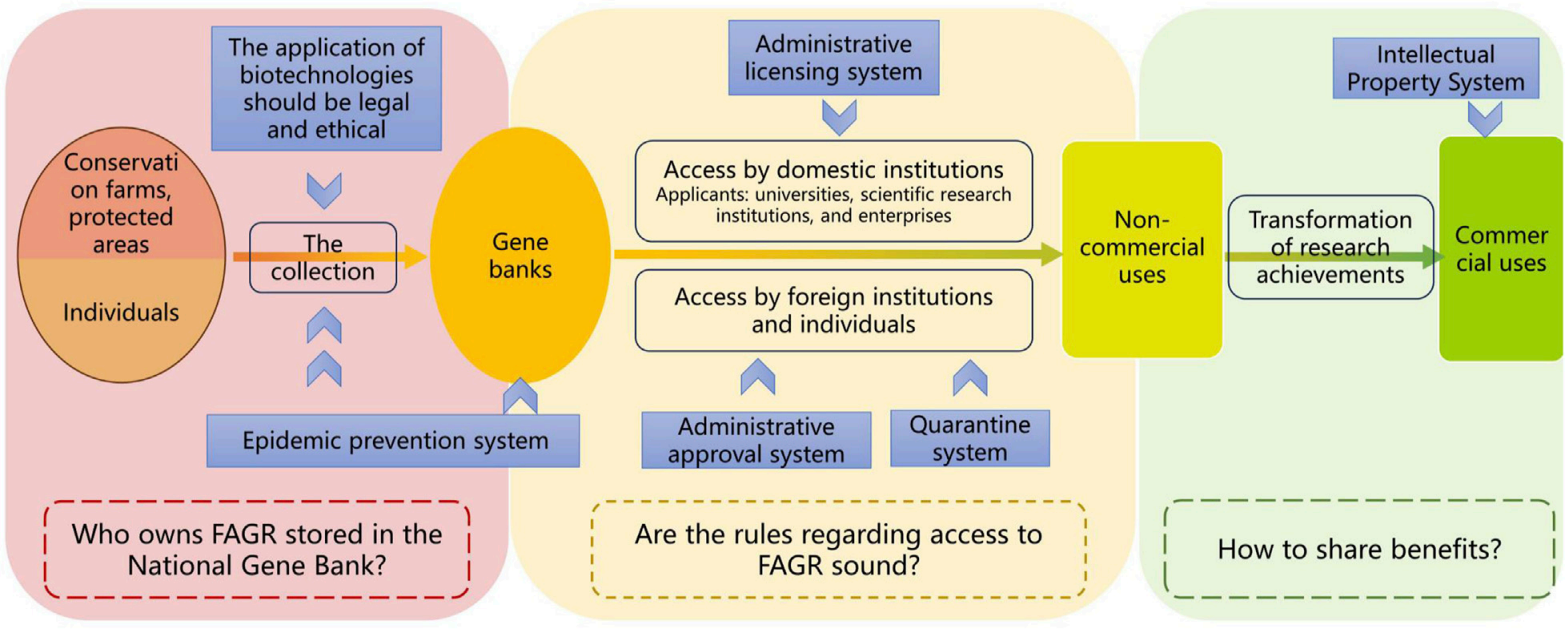
Current status of Chinese legal rules on collection, storage, and utilization of FAGR. The blue square represents the legal system that specific processes or entities must comply with. The dashed box represents questions raised through reflection on the existing legal rules. FAGR: farm animal genetic resources.

The operation of CNGBFs involves the collection, storage and utilization of FAGR. This paper will present the current status of Chinese legal rules regarding the collection, storage and utilization of FAGR, as well as the common issues involved in these processes (see Figure 4).

### 5.1 Collection

The *Animal Husbandry Law* requires CNGBFs to collect FAGR included in the *National Breed List of FAGR* [Article 14 (1)], covering both live animals and *in vitro* genetic materials. The collection of FAGR requires the application of specific reproductive and molecular biotechnologies, such as artificial insemination, embryo transfer, *in vitro* fertilization, and cloning (FAO, 2015). Chinese laws impose certain restrictions on the application of biotechnology. Chinese *Biosecurity Law* (The Standing Committee of the National People’s Congress, 2020a), stipulates that biotechnology applications should comply with ethical principles [Article 34 (2)]. According to this provision, technicians engaged in the collection of FAGR must pay particular attention to safeguarding animal welfare, ensuring that the process minimizes the suffering of animals.

### 5.2 Storage

The storage of FAGR by CNGBFs involves the ownership issue of collected FAGR. The provisions of multiple Chinese laws concern this issue, but they are not specific enough. For example, the *Animal Husbandry Law* stipulates that the nation holds sovereignty over FAGR (Article 17). This does not mean that the nation has the ownership of FAGR. The unclear ownership will affect the utilization of FAGR stored in gene banks. This paper will further discuss the ownership issue in Part 6.

### 5.3 Utilization

#### 5.3.1 Access to FAGR by domestic institutions

Chinese laws establish the following rules:

Firstly, users and utilization of FAGR: the *Animal Husbandry Law* does not directly specify who can use FAGR stored in CNGBFs and what activities FAGR stored in CNGBFs can be used for. Only paragraph 3 of Article 10 of the *Animal Husbandry Law* stipulates that the nation encourages and supports universities, research institutions, and enterprises to use FAGR for basic research. According to this provision, it is clear that eligible users of FAGR stored in CNGBFs include universities, research institutions, and enterprises, but only limited to basic research. The law does not provide a legal basis for individual access and commercial uses. However, in the international community, taking the Netherlands Gene Bank and the United States Gene Bank as examples, their utilization procedures are quite flexible and do not exclude these two types of usage^7^. As international research cooperation progresses, China needs to examine this difference.

Secondly, administrative licensing before utilizing FAGR: the *Animal Husbandry Law* contains restrictive provisions on the handling of stored FAGR by gene banks financially supported by central or provincial governments. It requires that before handling conserved FAGR, an approval should be obtained from the Ministry of Agriculture and Rural Affairs or the agricultural and rural affairs departments of governments at the provincial level [Article 14 (2)]. While this requirement ensures the safety of utilization, it also leads to an increase in administrative burdens for access. This is not conducive to the effective utilization of FAGR in gene banks.

Thirdly, informed consent prior to the utilization of FAGR: this requirement is stipulated in Chinese local laws or regulations, but there are no relevant provisions at the national legislative level in China. Nevertheless, at the local legislative level, there are inconsistencies in the scope of subjects of informed consent [see Articles 10 (1) and 20 (4) of the Administration Measures for the Access to and Benefit-Sharing of Biological Genetic Resources and Their Associated Traditional Knowledge of the Guangxi Zhuang Autonomous Region (for Trial Implementation) (Department of Ecology and Environment of Guangxi Zhuang Autonomous Region, 2021); Article 23 (2) of the Regulations on the Biodiversity Conservation in the Xiangxi Tujia and Miao Autonomous Prefecture of Hunan Province (The Standing Committee of People’s Congress of the Xiangxi Tujia and Miao Autonomous Prefecture, 2020)]. Informed consent is concerned with safeguarding the rights of relevant individuals. Its scope also affects the efficiency of utilizing genetic resources in gene banks. Therefore, the national legislation needs to properly set the scope of informed consent subjects in future.

#### 5.3.2 Access to FAGR by foreign institutions and individuals and international research collaborations

China has established the principle of national sovereignty over FAGR (Article 17 (1) of the Animal Husbandry Law; Article 53 (2) of the Biosecurity Law). Based on this, the *Animal Husbandry Law* stipulates that before transferring FAGR outside China, an approval should be obtained from the Ministry of Agriculture and Rural Affairs [Article 17 (1)]. In addition, according to Chinese *Biosecurity Law*, foreign institutions and individuals who intend to acquire FAGR within the territory of China should obtain the approval [see Article 58 (2)]. The *Animal Husbandry Law* also specifically provides for international research collaborations. The *Animal Husbandry Law* stipulates that before collaborating with foreign institutions or individuals in China to utilize FAGR included in the *National Breed List*, the approval shall be obtained from the Ministry of Agriculture and Rural Affairs [Article 17 (1)]. Moreover, the *Animal Husbandry Law* prohibits the export of newly discovered FAGR that have not been identified by the National Commission for FAGR, as well as the cooperative research and utilization of such resources with foreign institutions or individuals [Article 17 (3)].

### 5.4 Common issues

#### 5.4.1 Issues relating to intellectual property rights

Intellectual property issues are involved in the collection, storage and utilization of FAGR.

Gene banks for FAGR collect and store live animals or non-living genetic materials, including semen, embryos, eggs, DNA, and blood samples, etc. During these processes, live animals or non-living genetic materials cannot be patented. Chinese *Patent Law* (the Standing Committee of the National People’s Congress, 2020c) specifies that patents cannot be granted for animal breeds (Article 25), which include individual animals, reproductive cells, fertilized eggs, embryos, and so forth [Section 9.1.2.3 in chapter 10 of part 2 of Chinese Guidelines for Patent Examination (2023 edition) (China National Intellectual Property Administration, 2023)]. The reason why China excludes animal breeds from the scope of patent authorization is that animals are not products of artificial invention and that granting patent rights to animal breeds might raise ethical issues and hinder agricultural and scientific research (Wang, 2016). Therefore, live animals as well as semen, eggs, and embryos stored in gene banks for FAGR cannot be patented. Although genes or DNA fragments stored in gene banks do not fall within the scope of animal breeds, they are not patentable as well. Because genes or DNA fragments in natural form belong to scientific discoveries and cannot be granted for patent rights [Article 25 of Chinese Patent Law; section 9.1.2.2 in chapter 10 of part 2 of Chinese Guidelines for Patent Examination (2023 edition)]. However, there is an exception, namely, if the biological functions of the gene can be accurately characterized and industrial utilization values can be discovered, then the gene is patentable [section 9.1.2.2 in chapter 10 of part 2 of Chinese Guidelines for Patent Examination (2023 edition)]. At the storage phase, biological functions of genes or DNA fragments in gene banks cannot be accurately characterized. Consequently, genes or DNA fragments stored in gene banks are ineligible for patent protection.

FAGR stored in CNGBFs can be utilized in three ways: firstly, restoring endangered or extinct animal breeds; secondly, breeding for new animal breeds that have commercial value; and thirdly, identifying genetic variations that are valuable for breeding. Under Chinese intellectual property law framework, these three utilization scenarios will generate the following intellectual property rights: (1) Patent rights. There are two specific scenarios involved. The first scenario is whether the method of producing animal breeds is patentable. Although Chinese *Patent Law* stipulates that patent rights shall not be granted for animal breeds (Article 25), but it allows patent rights to be granted for non-biological production methods of animal breeds [Section 4.4 in chapter 1 of part 2 of Chinese Guidelines for Patent Examination (2023 edition)]. The second scenario is whether a gene is patentable. As mentioned above, if genetic variations with breeding value are discovered, the gene itself is eligible for patent protection [See section 9.1.2.2 in chapter 10 of part 2 of Chinese Guidelines for Patent Examination (2023 edition)]. (2) Copyright. If the discovery of genetic variations with breeding value is presented as a work, it can be protected by copyright [See Articles 2 and 3 of Chinese Copyright Law (the Standing Committee of the National People’s Congress, 2020b)]. (3) Geographical indications and trademarks. Gene banks for FAGR can be used to reconstruct livestock or poultry breeds in a specific region. If the reconstructed breed has unique qualities ascribed to the original environment, it can obtain the protection of geographical indication. China has formulated the *Administration Measures for the Geographical Indications of Agricultural Products* (Chinese Ministry of Agriculture and Rural Affairs, 2019b), which makes special provisions for the use of geographical indications for agricultural products. In addition, according to the provisions of Chinese *Trademark Law* (the Standing Committee of the National People’s Congress, 2019), geographical indications can also be applied for registration as certification trademarks or collective trademarks (Articles 3 and 13). (4) Trade Secrets. For newly developed livestock and poultry breeds with commercial value, the breeding institution or breeder can also protect the technical information in the breeding process via trade secrets. (5) It should be pointed out that similar to most countries, China has not established animal breed rights. This is taking into account the lack of genetic stability in animals compared to plant varieties (Lesser, 1993; Temmerman, 2011). However, as a signatory to the *TRIPS Agreement* and the *International Convention for the Protection of New Varieties of Plants* (International Union for the Protection of New Varieties of Plants, 1961), China has established the exclusive right for new varieties of plants through formulating the *Regulations on the Protection of New Varieties of Plants* (the State Council of China, 2014).

#### 5.4.2 Issues on benefit-sharing

At the national legislative level in China, there are no specific provisions on the sharing of benefits arising from utilizing FAGR stored in CNGBFs, while it does not prohibit benefit-sharing. The national legislation in China mainly focuses on the issue of benefit-sharing in scenarios involving exporting FAGR to foreign countries and international research cooperations. In this regard, the *Animal Husbandry Law* requires domestic individuals or institutions to propose a national benefit-sharing plan [Article 17 (1)]. However, the *Animal Husbandry Law* does not specify the basic content of the “national benefit-sharing plan”, such as the scope and form of benefit-sharing. Moreover, Chinese *Biosecurity Law* stipulates that when utilizing Chinese biological resources to conduct international research collaborations, it should be ensured that Chinese institutions and their researchers participate in the research process in a substantive manner [Article 59 (2)]. This requirement can be regarded as a form of non-monetary benefit-sharing.

Notably, although national legislation does not provide for the sharing of benefits arising from utilizing FAGR stored in CNGBFs, some local laws provide detailed provisions on this issue. The *Administration Measures for the Access to and Benefit-Sharing of Biological Genetic Resources and Their Associated Traditional Knowledge of the Guangxi Zhuang Autonomous Region (for Trial Implementation)* lays down the subject, scope, and form of benefit-sharing. The *Measures* stipulates that the monetary and non-monetary benefits arising from the utilization of biological genetic resources and the subsequent commercialization of research achievements shall be shared with the holders of biological genetic resources [see Article 10 (1)]. Similarly, the *Regulations on the Biodiversity Conservation in the Xiangxi Tujia and Miao Autonomous Prefecture* of Hunan Province also specifies the subject, scope and form of benefit-sharing [see Article 26 (3)]. However, the *Regulations* grants the providers of biological genetic resources and related traditional knowledge the right to benefit-sharing, which differs from the above-mentioned *Measures* of Guangxi.

#### 5.4.3 Issues pertaining to epidemic prevention for FAGR and public health

The processes of collecting, transporting, storing, importing from abroad, and transferring FAGR abroad involve the issue of controlling infectious diseases of livestock and poultry.

The collection of FAGR involves epidemic prevention for the animals from which samples are taken. The *Animal Husbandry Law* stipulates the epidemic prevention requirements for the entities engaged in the production and operation of stock animals, as well as genetic materials such as eggs, semen, and embryos (Articles 24 and 25). Articles 6 and 7 of the *Measures for the Examination of Animal Epidemic Prevention Requirements* (Chinese Ministry of Agriculture and Rural Affairs, 2022a) further elaborate on these requirements in detail. Moreover, the *Animal Husbandry Law* sets forth the requirement that donor animals for producing animal genetic materials should meet the national quality standards for healthy stock animals (Article 25).

Regarding the epidemic prevention during the transportation of FAGR, Chinese *Animal Epidemic Prevention Law* (the Standing Committee of the National People’s Congress, 2021b) establishes a system of quarantine and certification for animals and animal products [see Articles 29, 49, 51; See also Articles 8, 18, 39 of the Measures for the Quarantine of Animals (Chinese Ministry of Agriculture and Rural Affairs, 2022b)] as well as the epidemic prevention system for animal vehicles [Article 27 (1)].

Chinese *Animal Epidemic Prevention Law* also prescribes the epidemic prevention requirements for the storage of animal products. According to this *Law*, storage facilities for animal products shall carry out animal epidemic prevention procedures such as immunization, disinfection, diagnosing, isolation, purification, elimination, and harmless disposal (Article 7).

With respect to importing or exporting FAGR from or to abroad, the *Animal Husbandry Law* provides that such activities shall comply with the relevant procedures and quarantine measures stipulated in Chinese *Law on the Import and Export Animal and Plant Quarantine* [see Articles 16(1), 17(2)] (the Standing Committee of the National People’s Congress, 2009).

## 6 Strategic thoughts on improving legal rules for CNGBFs

Based on the above analysis, this paper brings up seven problems existing in the current legal rules for CNGBFs, which can be summarized as two institutional problems, four problems in the practical operation of CNGBFs, and one new issue emerging under the background of digitization. Two institutional problems are listed as follow. Firstly, regarding the fundamental legislation in the field of FAGR conservation, the utilization function of CNGBFs is not clear. Secondly, in terms of the administrative management, the coordination mechanism among multiple administrative authorities has not been established. The four key issues in practical operation of CNGBFs are: the ownership of FAGR in CNGBFs is not clear; domestic and international access rules are inadequate; and the rules of benefit-sharing are not constructed. The new issue arising under the background of digitization is the imperfect management system for digital sequence information (DSI).

### 6.1 Improving the basic legislation in the field of FAGR conservation: suggestions on clarifying the utilization function of CNGBFs

The conservation function of CNGBFs has been specified in the basic legislation in the field of FAGR conservation – the *Animal Husbandry Law*. However, regarding the utilization function of CNGBFs, the *Animal Husbandry Law* only stipulates that the nation encourages and supports universities, research institutions, and enterprises to use FAGR for basic research [Article 10 (3)]. Although it can be inferred from this provision that CNGBFs have utilization function, the *Animal Husbandry Law* does not make specific provisions for this function. To ensure the reasonable utilization of FAGR stored in CNGBFs, China should clarify the utilization function of CNGBFs, thereby better protecting the diversity of FAGR and promoting the development of breeding and genetics research. Specifically, Article 14 of the *Animal Husbandry Law* should clarify the obligation of CNGBFs to provide access to FAGR.

In addition, the *Global Plan* requires countries to take measures to support local production systems so as to protect, conserve, and develop animal genetic diversity (FAO, 2007a). In China, the main legal system related to this requirement is the *Intangible Cultural Heritage Law* (the Standing Committee of the National People’s Congress, 2011). Although the protection system established by this law can protect the diversity of FAGR to a certain extent, indigenous animals and associated traditional knowledge may still face the risk of loss. Because animals raised by local communities are vulnerable to changes in market demand (FAO, 2009). Since CNGBFs store a wealth of genetic resources, it can promptly provide breed introduction to local communities. Through breed introduction, lost indigenous animals and relevant traditional knowledge can be protected, maintained, and developed. Therefore, utilizing gene banks can provide better protection for endangered indigenous animals. The *Animal Husbandry Law* does not make specific provisions for conserving indigenous FAGR. To emphasize the importance of that aspect, this paper suggests that Article 14 of the *Animal Husbandry Law* should make a separate provision specifically addressing the obligation of CNGBFs to provide local communities with FAGR.

Furthermore, the laws do not define the roles of CNGBFs, that is, to clarify which CNGBFs are responsible for providing genetic resources to the public and which are responsible for preservation without making them available to the public. The absence of such provisions may hinder the sustainable utilization of FAGR. This article suggests that the supporting regulations of the Animal Husbandry Law, namely, the *Administration Measures for the Conservation Farms, Protected Areas, and Gene Banks for FAGR* should make provisions on this point.

### 6.2 Improving administrative management mechanism: a suggestion on enhancing the role of the National Commission for FAGR

The conservation and utilization functions of CNGBFs need to be implemented through the supervision of administrative departments. The *Administration Measures for the Conservation Farms, Protected Areas, and Gene Banks for FAGR* establishes that CNGBFs are administrated by the Ministry of Agriculture and Rural Areas. However, in reality, the administrative management of CNGBFs involves multiple departments, including the Ministry of Ecology and Environment, the National Intellectual Property Administration, the National Health Commission, and so forth. China should establish a mechanism to promote inter-departmental collaboration. In this regard, the *Animal Husbandry Law* may consider granting the National Commission for FAGR the legal status of coordinating relevant administrative departments while preserving the existing power of the National Commission for FAGR.

### 6.3 Improving ownership rules: suggestions on clarifying that FAGR in CNGBFs belong to the state

As we all know, ownership is a concept in civil law. Therefore, the issue of ownership of FAGR is directly related to how these genetic resources will be accessed and utilized in the future. Generally, livestock and poultry are considered as private property (Blackburn and Boettcher, 2010). Therefore, in practice, the ownership of FAGR stored in gene banks is often determined by negotiation between the gene bank and the original provider. The gene bank needs to negotiate with the original provider on whether to transfer the ownership of FAGR (FAO, 2012).

Chinese laws do not make specific provisions on the ownership of FAGR in CNGBFs. In China, the genetic materials in gene banks primarily originate from conservation farms and protected areas (Chinese Ministry of Agriculture, 2011; Chinese Ministry of Agriculture and Rural Affairs, 2019a). Of course, it is not ruled out that under special circumstances, genetic materials may be collected from private individuals. Article 14 (3) of the *Animal Husbandry Law* states that “relevant institutions and individuals shall cooperate with the gene bank for FAGR in collecting FAGR”. To some extent, this indicates a tendency that the *Animal Husbandry Law* supports the state ownership of FAGR. Considering that CNGBFs are public welfare and strategic infrastructures funded by the central government, the *Animal Husbandry Law* should stipulate that the ownership of FAGR stored in CNGBFs belongs to the nation. As a result, access to FAGR, as well as utilization beyond the scope of the initial consent, will require prior consent from the state, the owner of FAGR in CNGBFs. Since the nation is an abstract concept, the *Animal Husbandry Law* should further clarify the institution representing the nation in exercising ownership, so as to facilitate access and benefit-sharing practices.

### 6.4 Improving domestic access rules: suggestions on expanding the scope of subjects acquiring FAGR and types of use, as well as modifying the requirement of administrative licensing

First, expanding the scope of subjects acquiring FAGR. As mentioned above, individuals are not included in the scope of users in the Animal Husbandry Law. The conservation policy of FAGR in China has always emphasized “diversified sharing, development, and utilization” (Chinese Ministry of Agriculture and Rural Affairs, 2021b). In this sense, Article 10 (3) of the *Animal Husbandry Law* should clearly include individuals within the scope of users. This can also provide a legal basis for smallholder farmers to utilize FAGR to save endangered species.

Second, the legitimacy of commercial uses should be further clarified. Based on different purposes, utilization activities can be categorized into non-commercial research uses and commercial uses. As mentioned above, the legal basis of commercial uses is still lacking. Given that the distinction between commercial and non-commercial acquisition is not always clear^8^, it is necessary for China to provide a legal basis for commercial uses and actively develop relevant rules for them such as the conditions, procedures, and agreements for access to FAGR, etc. This will benefit animal breeding.

It is worth noting that Article 10 (3) of the *Animal Husbandry Law* uses the term “basic research”. This means that FAGR in CNGBFs can only be used for basic research. However, the current trend of scientific and technological development indicates that the boundary between basic research and applied research is becoming increasingly blurred^9^. In this sense, the *Animal Husbandry Law* should revise the term “basic research” by deleting the limitation of “basic”, so as to expand the types of uses. This is also consistent with the legislative purpose of the Animal Husbandry Law. One of the legislative purposes is to “cultivate excellent livestock and poultry breeds and revitalize the livestock and poultry breeding industry”. Broadly allowing the use of FAGR will help achieve this goal.

Third, modifying the requirement of administrative licensing. As mentioned above, access by domestic institutions must obtain approved from the Chinese Ministry of Agriculture and Rural Affairs of the State Council. This would result in increased administrative burdens for accessing FAGR, which in turn could potentially restrict the utilization of FAGR stored in CNGBFs. More importantly, if the utilization of FAGR is controlled through the mode of administrative licensing, it will conflict with the mode of ownership. In order to achieve a balance between efficiency and safety, China should, on the one hand, abolish the requirement of administrative licensing and adopt the mode of ownership; on the other hand, it should actively formulate basic standards for applying for the utilization, and model contracts for the transfer of genetic materials.

### 6.5 Improving international access rules: suggestions on clarifying the legal status of commercial uses

Similar to the provisions on domestic access, the *Animal Husbandry Law* also allows access by foreign institutions and individuals for non-commercial purposes, but it does not make clear provisions for commercial uses. Unlike domestic access, the *Animal Husbandry Law* clearly states that foreign individuals can be one of the subjects acquiring for FAGR (Article 17). To promote international cooperation, China should further clarify the legal status of commercial uses by foreign institutions and individuals and develop access procedures.

### 6.6 Improving legal rules on benefit-sharing: suggestions on systematically constructing the rules of benefit-sharing

There is a lack of systematic regulations on benefit-sharing at the Chinese national legislative level. It should actively draw upon the *CBD* and the *Nagoya Protocol* to systematically construct rules for benefit-sharing. In this regard, special attention should be paid to the following points: first, the scope of benefit-sharing should include both the benefits arising from the commercial utilization of FAGR and non-commercial research on FAGR. Second, benefits can be both monetary and non-monetary. Third, in the process of benefit-sharing, in addition to the state, which is the owner of FAGR, local communities should also be included as the main beneficiaries of benefit-sharing. Fourth, flexible methods of benefit-sharing should be adopted. Users should be allowed to make agreements with the beneficiaries on matters related to benefit-sharing, as long as such agreements do not violate legal provisions. Fifth, Chinese *Patent Law* establishes a system for disclosing sources of genetic resources [Article 26 (5)], but it does not set corresponding legal responsibilities. This may lead to false disclosures. The *Patent Law* should clearly stipulate the legal consequences of falsely disclosing the source of genetic resources, thereby enhancing the support mechanism for benefit-sharing implementation.

### 6.7 Embracing the challenges of the big data era: suggestions on improving the management system of DSI

With the rapid development of digital technology and information technology, digital sequence information (DSI) on genetic resources has emerged. DSI can contribute to scientific research as well as to the development of biodiversity, food security, and human, animal and plant health (Conference of the Parties to the Convention on Biological Diversity, 2018). However, the emergence of DSI has changed the reliance on access to tangible genetic resources, posing a challenge to the access and benefit-sharing regime for genetic resources established by the *CBD* and the *Nagoya Protocol* (Subsidiary Body on Scientific, Technical and Technological Advice, 2018). In December 2016, the DSI issue was discussed for the first time at the 13th Conference of the Parties to the *CBD* (Conference of the Parties to the Convention on Biological Diversity, 2016) and the second Meeting of the Parties to the *Nagoya Protocol* (Conference of the Parties to the Convention on Biological Diversity Serving as the Meeting of the Parties to the Nagoya Protocol, 2016)^10^. By December 2022, the 15th Conference of the Parties to the *CBD* had reached a preliminary consensus on the sharing of benefits arising from the utilization of DSI (Conference of the Parties to the Convention on Biological Diversity, 2022). Nevertheless, there are still disagreements on issues such as the concept and scope of DSI, as well as domestic measures for access and benefit-sharing (see Ad Hoc Technical Expert Group on Digital Sequence Information on Genetic Resources, 2020b; Ad Hoc Technical Expert Group on Digital Sequence Information on Genetic Resources, 2020a). As a party to the *CBD* and the *Nagoya Protocol*, China needs to amend its *Agricultural Law* (the Standing Committee of the National People’s Congress, 2012a), *Seed Law* (the Standing Committee of the National People’s Congress, 2021c), *Animal Husbandry Law* and other laws in a timely manner in order to positively respond to the challenges posed by DSI.

In improving relevant legal rules, China should focus on the following three aspects: first, DSI involves not only the issues of access and benefit-sharing under the *CBD* and the *Nagoya Protocol*, but also data security issues under Chinese *Data Security Law* (the Standing Committee of the National People’s Congress, 2021a). Therefore, regarding the access policy for DSI, China should clarify the classification and categorization of DSI, and set different access policies for DSI based on different risk levels, so that the access policy can both promote open sharing and ensure national security. Second, a specialized national legislation on DSI should be formulated to provide a legal basis for DSI access and benefit-sharing. This specialized legislation should clarify issues such as the definition, scope, classification, acquisition rules, as well as benefit-sharing of DSI. The formulation of specialized legislation can not only stipulate the common issues related to DSI across various field, but also address the unique issues of DSI that are different from tangible genetic resources, such as the need for flexibility in access policies. Third, Chinese *Biosecurity Law* establishes the biosecurity system. With the development of technology, the application of DSI in synthetic biology may pose challenges to Chinese biosecurity. In this sense, Chinese *Biosecurity Law* should include DSI within its regulatory scope. Specifically, the *Biosecurity Law* can include the concept of biological resources in its supplementary provisions section (pertaining to the explanation of terms meanings), and specify that the scope of this concept includes DSI.

## 7 Conclusion

CNGBFs play an important role in FAGR conservation, animal husbandry research, and animal breeding. In order to regulate the management and operation of CNGBFs, Chinese *Animal Husbandry Law* and the *Administration Measures for the Conservation Farms, Protected Areas, and Gene Banks for FAGR* specifically establish a set of rules. Besides, other sources of law in China also cover rules related to the management and operation of CNGBFs. However, these rules have shortcomings, which can be summarized as two institutional problems, four key problems in practical operation, and one new issue emerging under the background of digitization.

To fully realize the conservation and utilization functions of CNGBFs, this paper proposes directions for improving legal rules in response to these issues, as detailed below: (1) clarifying the utilization function of CNGBFs in the *Animal Husbandry Law*; (2) granting the National Commission for FAGR the legal status to coordinate multiple administrative departments; (3) clarifying that FAGR in CNGBFs belongs to the state in the *Animal Husbandry Law*, and the institution representing the nation in exercising ownership; (4) the *Animal Husbandry Law* should expand the scope of subjects acquiring FAGR and types of use, as well as modify the requirement of administrative licensing. Individual access and commercial uses should be permitted; (5) further clarifying the legal status of commercial access by foreign institutions and individuals in the *Animal Husbandry Law*; (6) systematically establishing benefit-sharing rules; (7) improving the management system of DSI.

## Data Availability

The original contributions presented in the study are included in the article/Supplementary Material, further inquiries can be directed to the corresponding authors.

## Footnotes

1 The Stock Animal Administration Regulations was abolished in 2018.

2 The Animal Husbandry Law has undergone two amendments, which were conducted on 24 April 2015 and 30 October 2022 respectively. The latest version of the Animal Husbandry Law took effect on 1 March 2023.

3 National conserved breeds are included in the National Breed List of FAGR, which is formulated by the Ministry of Agricultural and Rural Affairs of the State Council.

4 Provincial conserved breeds are included in the Provincial Breed List of FAGR, which is formulated by the agriculture and rural affairs departments of the people’s governments at the provincial level.

5 Please refer to the Supplementary Material for the specific laws formulated by these authorities.

6 In 2018, the State Council of China abolished the Ministry of Agriculture and established the Ministry of Agriculture and Rural Affairs.

7 Applications and issuance. https://www.wur.nl/nl/onderzoek-resultaten/kennisonline-onderzoeksprojecten-lvvn/centre-for-genetic-resources-the-netherlands/animal-genetic-resources/genebank-for-animal-resources/applications-and-issuance.htm (Access 16 August 2024); National Animal Germplasm Program. https://agrin.ars.usda.gov/germplasm_request_page_dev?language=EN&record_source=US (Access 16 August 2024).

8 Secretariat of the Convention on Biological Diversity (2011). Theme: Uses of genetic resources. https://www.cbd.int/abs/infokit/revised/web/factsheet-uses-en.pdf (Accessed 16 August 2024).

9 Blurring divides between basic and applied. Nat Food 2, 831 (2021). https://doi.org/10.1038/s43016-021-00414-6

10 Digital sequence information on genetic resources. https://www.cbd.int/dsi-gr
