# Supplementary material for: The current status and improvement directions of legal rules regarding Chinese national gene banks for farm animal genetic resources
Source: Front Genet. 2024 Oct 9;15:1413625. doi: 10.3389/fgene.2024.1413625 (PMC11496119; doi:10.3389/fgene.2024.1413625)
Supplement: Supplementary file 1 [file Table1.docx]

**Supplementary Material Table 1: The main laws enacted by the Chinese National People’s Congress and its Standing Committee**

| Number | Legislature | The laws | Year of Adoption/ Amendment |
| --- | --- | --- | --- |
| 1 | National People’s Congress | Civil Code of the People’s Republic of China | 2020 Adoption |
| 2 | The Standing Committee of the National People’s Congress | Animal Husbandry Law of the People’s Republic of China | 2022 Amendment |
| 3 | The Standing Committee of the National People’s Congress | Environmental Protection Law of the People’s Republic of China | 2014 Amendment |
| 4 | The Standing Committee of the National People’s Congress | Biosecurity Law of the People’s Republic of China | 2020 Adoption |
| 5 | The Standing Committee of the National People’s Congress | Agriculture Law of the People’s Republic of China | 2012 Amendment |
| 6 | The Standing Committee of the National People’s Congress | Law of the People’s Republic of China on Scientific and Technological Progress | 2021 Amendment |
| 7 | The Standing Committee of the National People’s Congress | Law of the People’s Republic of China on the Popularization of Agricultural Technology | 2021 Amendment |
| 8 | The Standing Committee of the National People’s Congress | Law of the People’s Republic of China on the Import and Export Animal and Plant Quarantine | 2009 Amendment |
| 9 | The Standing Committee of the National People’s Congress | Law of the People’s Republic of China on Prevention and Treatment of Infectious Diseases | 2013 Amendment |
| 10 | The Standing Committee of the National People’s Congress | Animal Epidemic Prevention Law of the People’s Republic of China | 2021 Amendment |
| 11 | The Standing Committee of the National People’s Congress | Patent Law of the People’s Republic of China | 2020 Amendment |
| 12 | The Standing Committee of the National People’s Congress | Copyright Law of the People’s Republic of China | 2020 Amendment |
| 13 | The Standing Committee of the National People’s Congress | Trademark Law of the People’s Republic of China | 2019 Amendment |
| 14 | The Standing Committee of the National People’s Congress | Intangible Cultural Heritage Law of the People’s Republic of China | 2011 Adoption |

**Supplementary Material Table 2: The main administrative regulations formulated by the State Council of China**

| Number | The administrative regulations | Year of Adoption/ Amendment |
| --- | --- | --- |
| 1 | Measures of the People’s Republic of China for the Examination and Approval of Import & Export and the Foreign Cooperative Research on the Application of Genetic Resources of Farm Animal | 2008 Adoption |
| 2 | Regulations for the Implementation of the Patent Law of the People’s Republic of China | 2023 Amendment |

**Supplementary Material Table 3: The main administrative regulations formulated by departments under the State Council of China**

| Number | Legislature | The administrative regulations | Year of Adoption/ Amendment |
| --- | --- | --- | --- |
| 1 | Chinese Ministry of Agriculture^[[1]](#footnote-1)^ | Measures for the Conservation Farms, Protected Areas, and Gene Banks for Farm Animal Genetic Resources | 2006 Adoption |
| 2 | Chinese Ministry of Agriculture and Rural Affairs | Administration Measures for the Geographical Indications of Agricultural Products | 2019 Amendment |
| 3 | Chinese Ministry of Agriculture and Rural Affairs | Measures for the Quarantine of Animals | 2022 Adoption |
| 4 | Chinese Ministry of Agriculture and Rural Affairs | Measures for the Examination of Animal Epidemic Prevention Requirements | 2022 Adoption |

**Supplementary Material Table 4: The main local laws and regulations formulated by local legislatures that possess legislative power in accordance with the provisions of the Legislation Law of the People’s Republic of China**

| Number | Legislature | The local laws and regulations | Year of Adoption/ Amendment |
| --- | --- | --- | --- |
| 1 | Department of Ecology and Environment of Guangxi Zhuang Autonomous Region | Administration Measures for the Access to and Benefit-Sharing of Biological Genetic Resources and Their Associated Traditional Knowledge of the Guangxi Zhuang Autonomous Region (for Trial Implementation) | 2021 Adoption |
| 2 | The Standing Committee of the Shandong Provincial People’s Congress | Regulations on the Biodiversity Conservation in Shandong Province | 2023 Adoption |
| 3 | The Standing Committee of the Yunnan Provincial People’s Congress | Regulations on the Biodiversity Conservation in Yunnan Province | 2019 Adoption |
| 4 | The Standing Committee of the Xiangxi Tujia and Miao Autonomous Prefecture People’s Congress | Regulations on the Biodiversity Conservation in the Xiangxi Tujia and Miao Autonomous Prefecture | 2020 Adoption |

1. In 2018, the State Council of China abolished the Ministry of Agriculture and established the Ministry of Agriculture and Rural Affairs. [↑](#footnote-ref-1)
